# A93 IMPROVING INPATIENT ENDOSCOPY PROCEDURAL THROUGHPUT: A QUALITY IMPROVEMENT INITIATIVE AT AN ACADEMIC MEDICAL CENTER

**DOI:** 10.1093/jcag/gwac036.093

**Published:** 2023-03-07

**Authors:** M Alsager, D Hudson, Z hindi, C Lavalle, V Iablokov, A Alajmi, N khanna, K qumosani, M brahmania

**Affiliations:** Western, London, Canada

## Abstract

**Background:**

Currently, due to pandemic-related supply and personnel constraints, increased strain is being placed on inpatient endoscopic services. At our academic center, inpatient procedural throughput/efficiency is only 65%, which is leading to excess procedures being completed on-call or on weekends.

**Purpose:**

Our quality improvement study attempted to improve throughput of the inpatient endoscopy suite by 1 additional procedure completed per day, over a period of 12 months of study.

**Method:**

An interprofessional team of gastroenterology fellows, attending physicians, nurses and nurse managers was created to investigate throughput concerns. Baseline data was collected via direct observation and completion of a time study over a period of 1-2 months.

Subsequently, a process flow diagram was completed. A combination of root cause analysis tools (ie. Stakeholder interviews, Pareto chart and Driver Diagram were then utilized to identify areas for improvement. A delay in procedural start time was identified as a strong culprit for reduced patient throughput. Potential interventions proposed included: early physician handover start time, constructing a standardized patient procedure list, and improving timeliness of patient transfer to the endoscopy suite.

**Result(s):**

**Stakeholder Interview(s): Completed via Anonymous Survey/Google Forms:**

**See:**

https://docs.google.com/forms/d/e/1FAIpQLSe-PDL31ClU8g-BChdBoBYhPoBhGBZVmlToTTZJnYKlEHz-GQ/viewform?usp=sf_link

Concerns:

- Inpatient procedure list not consistently being completed prior to starting endoscopy

- Physician handover in the morning can cause significant delays in starting inpatient endoscopy

- Concern patient/porter transfer related delays

**Conclusion(s):**

Our current data identifies that the most effective intervention included developing a standardized procedure list and mandating the first case is an EGD, minimizing delay due to inadequate bowel preparation. Concordantly, arranging timely patient transfer to the endoscopy suite, thereby minimizing delays due to patient portering services, was also found to be effective. Our average procedural time for completion of an EGD and associated recovery is approximately 25-30 minutes. After multiple interventions/PDSA cycles we obtained a more optimized procedural suite start time of 08:22 am, on average, which resulted in the completion of an additional procedure and increased the throughput of our endoscopy unit.

**Please acknowledge all funding agencies by checking the applicable boxes below:**

None

**Disclosure of Interest:**

None Declared

